# Supplementary material for: Clinical trials in depression: Integrated collection across EU and US registries
Source: Wellcome Open Res. 2026 May 7;10:670. Originally published 2025 Dec 10. [Version 2] doi: 10.12688/wellcomeopenres.25126.2 (PMC13049435; doi:10.12688/wellcomeopenres.25126.2)
Supplement: Peer review history

# Reviewer report: Reviewer response for version 1

**DOI:** 10.21956/wellcomeopenres.27699.r150766

**Published:** 2026-04-03

**Authors:** Cedric Lemarchand

**Affiliations:** 1 University of Rennes (Ringgold ID: 27079), Rennes, Britanny, France

This review sets a high standard for future articles in this field. The following suggestions are therefore minor:

 1/ The distinction between NMDA antagonists and psychedelics is particularly relevant, given the recent tendency to present psychedelics as a homogeneous group. However, a few clarifications could strengthen this aspect:

 - It would be helpful to further elaborate on the distinction between these two classes and to provide examples of molecules included in each category, even if this information is already provided in the supplementary material.

 - The authors may also wish to justify this classification choice over a more holistic "psychedelic" approach, which remains common in the synthesis literature(1), and to explain why future studies might benefit from adopting a similar framework.

 While such a discussion may seem unexpected in a data note, the conceptual ambiguity in this field makes it valuable.

 2/ There is also room for a more in-depth discussion of the growing interest in NMDA antagonists and psychedelics, with ketamine and psilocybin leading their respective classes in the treatment of depression. Both appear to follow similar trajectories, with hallucinogens as a whole often being presented as game changers.

 In addition, the manuscript could further address the methodological challenges specific to hallucinogens, particularly functional unblinding. These substances compromise blinding procedures, as participants are often able to guess which treatment they have received. Moreover, trial registrations are often incomplete or insufficiently detailed. Even in the context of a data note, a brief mention of the observed discrepancies in trial registrations would be relevant(2).

 3/ Further development on the severity of depression would also be valuable. One key issue lies in the definition of depression itself. For instance, ketamine and psilocybin have primarily been studied in the context of treatment-resistant depression (TRD), yet TRD remains a poorly standardized definition(3), which affects the interpretability and comparability of results.

 4/ The chosen time interval is another point that could be reconsidered. While the intention to be exhaustive is understandable, trials conducted prior to 2005 may not meet current standards for trial registration, as the International Committee of Medical Journal Editors Recommendations (ICMJE) policy came into effect that year(4). Moreover, earlier records are often less reliable and more prone to incomplete reporting. As a result, the current time interval makes Figure 7 difficult to interpret. A stronger focus on recent years, where most developments have occurred, would improve readability.

 5/ Finally, while the methodology is clearly described, it would be useful to know whether it has been validated to assess its sensitivity and precision. Demonstrating robust performance metrics would strengthen confidence in the approach and support its use in future studies.

Are sufficient details of methods and materials provided to allow replication by others?

Yes

Is the rationale for creating the dataset(s) clearly described?

Yes

Are the datasets clearly presented in a useable and accessible format?

Yes

Are the protocols appropriate and is the work technically sound?

Yes

Reviewer Expertise:

Public health, bibliometrics, methodology, clinical trials, hallucinogens

I confirm that I have read this submission and believe that I have an appropriate level of expertise to confirm that it is of an acceptable scientific standard.

## References

[1] : Psychedelic medicine: mechanisms, evidence, and translation to practice. *BMJ* .2026;392: 10.1136/bmj-2024-081723 10.1136/bmj-2024-081723 41730563

[2] : Consistency of protocol and safety data reporting in clinical trial registrations and corresponding publications of interventions involving MDMA and psilocybin. *Journal of Clinical Epidemiology* .2026;192: 10.1016/j.jclinepi.2026.112170 10.1016/j.jclinepi.2026.112170 41581835

[3] : Defining treatment‐resistant depression. *Depression and Anxiety* .2020;37(2) : 10.1002/da.22968 134-145 10.1002/da.22968 31638723

[4] : Clinical Trial Registration: A Statement from the International Committee of Medical Journal Editors. *New England Journal of Medicine* .2004;351(12) : 10.1056/NEJMe048225 1250-1251 10.1056/NEJMe048225 15356289

# Response

**Published:** 2026-04-24

**Authors:** Kate Stewart

**Affiliations:** King's College London Social Genetic & Developmental Psychiatry Centre, London, England, UK

We thank the reviewer for their positive feedback and constructive comments. Our responses to specific comments are outlined below:

1. ***Reviewer:*** *The distinction between NMDA antagonists and psychedelics is particularly relevant, given the recent tendency to present psychedelics as a homogeneous group. However, a few clarifications could strengthen this aspect:*

- *It would be helpful to further elaborate on the distinction between these two classes and to provide examples of molecules included in each category, even if this information is already provided in the supplementary material.*
- *The authors may also wish to justify this classification choice over a more holistic "psychedelic" approach, which remains common in the synthesis literature(1), and to explain why future studies might benefit from adopting a similar framework.*

*While such a discussion may seem unexpected in a data note, the conceptual ambiguity in this field makes it valuable.* **Response: **We thank the reviewer for this helpful insight. In the section ‘Data Summary, Derived dataset creation’, we have clarified our choice to distinguish between NMDA receptor antagonists and serotonergic psychedelics: “For the fast-moving field of psychedelic drugs, we chose to provide a high level of granularity when categorising, for example separating NMDA receptor antagonists and serotonergic psychedelics.” Although we felt that inclusion of specific molecules included in each category was beyond the scope of this data note, specific drugs included in each category of drug class is now outlined in Supplementary Table 5.

1. ***Reviewer:*** *There is also room for a more in-depth discussion of the growing interest in NMDA antagonists and psychedelics, with ketamine and psilocybin leading their respective classes in the treatment of depression. Both appear to follow similar trajectories, with hallucinogens as a whole often being presented as game changers.*

*In addition, the manuscript could further address the methodological challenges specific to hallucinogens, particularly functional unblinding. These substances compromise blinding procedures, as participants are often able to guess which treatment they have received. Moreover, trial registrations are often incomplete or insufficiently detailed. Even in the context of a data note, a brief mention of the observed discrepancies in trial registrations would be relevant(2).* **Response: **We thank the reviewer for this raising these issues. We acknowledge the methodological challenges that arise in clinical trials, such as functional unblinding, however we feel that they are outside of the scope of this data note. We agree that trial registrations can be incomplete or insufficiently detailed and have now noted in the limitations section the presence of post-registration changes in psychedelic clinical trials: “Trials are registered before data collection, and records may not be updated afterwards. This may be particularly relevant for recent trials in psychedelic drugs where post-registration changes appear common.”

1. ***Reviewer:*** *Further development on the severity of depression would also be valuable. One key issue lies in the definition of depression itself. For instance, ketamine and psilocybin have primarily been studied in the context of treatment-resistant depression (TRD), yet TRD remains a poorly standardized definition(3), which affects the interpretability and comparability of results.*

**Response: **We thank the reviewer for raising this important point on standardisation of definitions of depression severity and treatment resistant depression. We have noted this challenge in our limitations section and point to current work being undertaken to address this challenge: “Definitions of depression severity, and treatment-resistant depression (TRD), are not standardised, making comparison between studies challenging. Defining TRD and other relevant phenotypes is an ongoing area of work.”

1. ***Reviewer:*** *The chosen time interval is another point that could be reconsidered. While the intention to be exhaustive is understandable, trials conducted prior to 2005 may not meet current standards for trial registration, as the International Committee of Medical Journal Editors Recommendations (ICMJE) policy came into effect that year(4). Moreover, earlier records are often less reliable and more prone to incomplete reporting. As a result, the current time interval makes Figure 7 difficult to interpret. A stronger focus on recent years, where most developments have occurred, would improve readability.*

**Response: **We thank the reviewer for highlighting the challenges raised by including older records which may be less reliable and complete. We encourage future researchers to delve further into this area in order to better understand recent progress. We have chosen to retain our original timeframe as we feel this provides the benefits of a richer data source, however we have acknowledged this issue in our limitations section: “Changes in policy, such as the introduction of the International Committee of Medical Journal Editors Recommendations (ICMJE) in 2005 to require registration of clinical trials, will impact the standard of information reported within trial registration across the time period covered.”

1. ***Reviewer:*** *Finally, while the methodology is clearly described, it would be useful to know whether it has been validated to assess its sensitivity and precision. Demonstrating robust performance metrics would strengthen confidence in the approach and support its use in future studies.*

**Response: **We thank the reviewer for their feedback. Internal checks for completeness and accuracy were completed, however we did not undertake formal analysis of sensitivity and precision. We acknowledge other limitations within the data note, for example that only open access publications could be included.

# Reviewer report: Reviewer response for version 1

**DOI:** 10.21956/wellcomeopenres.27699.r147718

**Published:** 2026-02-26

**Authors:** Marin Jukic

**Affiliations:** 1 Karolinska Institutet, Solna, Sweden

This manuscript provides a valuable overview of the clinical trials conducted on depression. It offers an unbiased quantification of trial properties along with temporal trends. I have only a few minor remarks that could improve accuracy and readability.

 Please use "effectiveness" instead of "efficacy" where appropriate. Clinical trials usually address effectiveness (1).

 Figure 3. Distribution of trial intervention categories (n = 8,853) includes an "Other" category. Please describe what this term means in the figure legend.

 Figure 4. Distribution of drug interventions by antidepressant class (panel A) and antidepressant drug (panel B) should be presented as a table for greater clarity.

 To enhance clarity and readability, please provide a list explicitly stating which drug belongs to which class. This can be included in the text or in the figure legend, for example: Class 1 (Drug 1, Drug 2, Drug 3, ...), Class 2 (Drug 5, Drug 6, Drug 7, ...), Class 3, etc.

 The "Temporal trends in pharmacological research" section should be expanded. Please comment on the rapid increase in the number of SARI-based trials in recent years.

 The manuscript would benefit from the inclusion of a figure representing the Sponsor type (year) graph, primarily to show temporal trends in Healthcare versus Academic/Public distribution.

Are sufficient details of methods and materials provided to allow replication by others?

Yes

Is the rationale for creating the dataset(s) clearly described?

Yes

Are the datasets clearly presented in a useable and accessible format?

Yes

Are the protocols appropriate and is the work technically sound?

Yes

Reviewer Expertise:

Pharmacogenetics (CYP genotyping), Drug metabolism, Pharmacokinetics, and Therapeutic drug monitoring

I confirm that I have read this submission and believe that I have an appropriate level of expertise to confirm that it is of an acceptable scientific standard.

## References

[1] : The efficacy-effectiveness gap.2021; 10.1016/B978-0-12-817663-4.00024-6 9-19 10.1016/B978-0-12-817663-4.00024-6

# Response

**Published:** 2026-04-24

**Authors:** Kate Stewart

**Affiliations:** King's College London Social Genetic & Developmental Psychiatry Centre, London, England, UK

We thank the reviewer for their valuable feedback. We have implemented the following revisions in response to reviewer comments. Reviewer comments are italicised.

1. *Please use "effectiveness" instead of "efficacy" where appropriate. Clinical trials usually address effectiveness (1).*

**Response: **We have replaced “efficacy” with “effectiveness” in the introduction for accuracy: “Trials systematically assess the effectiveness and safety of antidepressant interventions over time, generating rich clinical datasets that may include genetic data, or samples from which genetic data can be generated.”

1. *Figure 3. Distribution of trial intervention categories (n = 8,853) includes an "Other" category. Please describe what this term means in the figure legend.*

**Response: **Thank you for this comment. The legend for Figure 3 now includes a descriptive sentence of what is included in the category “Other”: “The category ‘Other’ is chosen by the creator of the trial record. There are over 1000 interventions included in this category. A proportion of these interventions are listed as placebo, waitlist control or treatment as usual. Others are likely miscategorised due to human error (e.g. computer assisted CBT) or are non-specific (e.g. internet intervention). A proportion of interventions do not clearly fit into the pre-determined intervention categories (e.g. bilateral massage, diary).” For clarity, we have also added a sentence to the legend of Figures 5 and 8 to explain the definition of the category ‘Other’: “The category ‘Other’ was defined by analysts. The category ‘Unspecified’ indicates that the trial record listed a generic term e.g. antidepressant treatment. Drugs included in these categories can be found in Supplementary Table 5.”

1. *Figure 4. Distribution of drug interventions by antidepressant class (panel A) and antidepressant drug (panel B) should be presented as a table for greater clarity.*

**Response: **We have added two new tables to present the distribution of drug interventions by antidepressant class and antidepressant drug (please see Tables 5 and 6).

1. *To enhance clarity and readability, please provide a list explicitly stating which drug belongs to which class. This can be included in the text or in the figure legend, for example: Class 1 (Drug 1, Drug 2, Drug 3, ...), Class 2 (Drug 5, Drug 6, Drug 7, ...), Class 3, etc.*

**Response: **We have added a new table listing drugs to treat depression and their drug classes to Extended Data (please see Supplementary Table 5).

1. *The "Temporal trends in pharmacological research" section should be expanded. Please comment on the rapid increase in the number of SARI-based trials in recent years.*

**Response: **Thank you for this feedback. The section on temporal trends in pharmacological research has been expanded to include other drug classes: “There has also been an increase in clinical trials including serotonergic psychedelics since 2023. Trials including SARI, NRI, MAOI and tetracyclic drugs have remained low across this time period.”

1. *The manuscript would benefit from the inclusion of a figure representing the Sponsor type (year) graph, primarily to show temporal trends in Healthcare versus Academic/Public distribution.*

**Response: **We have added a new figure of temporal trends in sponsor type over time (please see Figure 4).
